# COVID-19 vaccine: Call for employees in international transportation industries and international travelers as the first priority in global distribution

**DOI:** 10.1515/med-2021-0210

**Published:** 2021-01-11

**Authors:** Zhuo Yu, Gang Wang, Emanuel Goldman, Barbara Zangerl, Ning Xie, Yanhong Cao, Jingyu Chen, Sara W. Day, Scott C. Howard, Marcello Maida, Kunal Ray, Monica M. Jablonski, Jiafu Ji, Arnold Postlethwaite, Weikuan Gu, Dianjun Sun, Lotfi Aleya

**Affiliations:** Heilongjiang Academy of Traditional Chinese Medicine, Sanfu Road 142, Xiangfang District, Harbin, Heilongjiang, 150040, People’s Republic of China; The First Affiliated Hospital of Harbin Medical University, 23 Youzheng Street, Nangang District, Harbin 150001, China; Department of Microbiology, Biochemistry and Molecular Genetics, New Jersey Medical School, Rutgers University, , Newark, NJ 07103, United States of America; School of Optometry and Vision Science, UNSW Sydney, Sydney, NSW, Australia; College of Business, University of Louisville, Louisville, KY 40292, United States of America; Center for Endemic Disease Control, Chinese Center for Disease Control and Prevention, Harbin Medical University; Key Laboratory of Etiologic Epidemiology, Education Bureau of Heilongjiang Province & Ministry of Health [23618104], 157 Baojian Road, Harbin, Heilongjiang, 150081, People’s Republic of China; Department of Chinese Medicine, First Clinical School of Medicine, Heilongjiang University Of Chinese Medicine, 24 Heping Rd, Xiangfang District, Harbin, Heilongjiang, 150040, China; College of Nursing, University of Tennessee Health Science Center, Memphis, TN 38105, United States of America; Gastroenterology and Endoscopy Unit, S. Elia-Raimondi Hospital, 93100, Caltanissetta, Italy; School of Biological Science, Ramkrishna Mission Vivekananda Education & Research Institute, Narendrapur 700103, West Bengal, India; Department of Ophthalmology, University of Tennessee Health Science Center, Memphis, Tennessee, 38163, United States of America; Beijing Cancer Hospital and Key Laboratory of Carcinogenesis and Translational Research, Department of Gastrointestinal Surgery, Peking University Cancer Hospital and Institute, Beijing 100142, People’s Republic of China; Department of Medicine, University of Tennessee Health Science Center, Memphis, Tennessee, 38163, United States of America; Research Service, Memphis VA Medical Center, 1030 Jefferson Avenue, Memphis, TN, 38104, United States of America; Department of Orthopedic Surgery and BME-Campbell Clinic, University of Tennessee Health Science Center, 956 Court Avenue, Memphis, Tennessee, 38163, United States of America; Chrono-Environnement Laboratory, UMR CNRS 6249, Bourgogne Franche-Comté University, Université de Franche-Comté 16, Route de Gray, F-25030, Besançon Cedex, France

**Keywords:** COVID-19, priority, international collaboration, international transportation, vaccine

## Abstract

While countries are in a hurry to obtain SARS-CoV-2 vaccine, we are concerned with the availability of vaccine and whether a vaccine will be available to all in need. We predicted three possible scenarios for vaccine distributions and urge an international united action on the worldwide equitable access. In case the international community does not reach a consensus on how to distribute the vaccine to achieve worldwide equitable access, we call for a distribution plan that includes the employees in international transportation industries and international travelers to halt the disease transmission and promote the recovery of the global economy.

We are glad to see “the joint appeal” by 3,000 people and the comment on the journal [1,2]. We all appreciate the effort of United Nation on mobilizing $28 billion needed to secure vaccines for all [3]. However, we are concerned about the challenge in the production as well as in the capability of distribution of the vaccine in time. While the world is hoping the availability of a vaccine by the end of the year and optimistic about vaccine for all, there are many obstacles in the timely distribution and equal availability to all in the world. Additionally, the anticipated increase in respiratory diseases with winter approaching in the northern hemisphere leads to serious concerns whether a vaccine will be available to all in time [4,5,6,7].

Even under the assumption that a COVID-19 vaccine will become available in time, prioritization regarding its distribution to 195 countries and maintenance of worldwide equitable access will remain challenging [8]. Notably a few countries, including China and the US, are predicted to have the best chance and greatest resources to produce the vaccine and, thus, are most likely to have access to immediate distribution. This implies that worldwide equitable access will not be accomplished if the supply of an approved SARS-CoV-2 vaccine is inadequate [9,10], leading to three possible scenarios. First, the vaccine may only be produced and distributed in the country of origin with possible distribution to a few countries that have close ties with the vaccine-producing country. In the second scenario, the vaccine is successfully developed and produced in several countries at approximately the same time and distributed in those countries, again, possibly including additional countries with close ties. The third scenario assumes that the vaccine is first produced in one or several countries, but the vaccine is supplied worldwide on the basis of the most pressing need. In Table 1, we summarized the impact of these different usages of the SARS-CoV-2 vaccine.

**Table 1:** Impact of different usages of vaccine

Types of effects	Scenario 1	Scenario 2	Scenario 3
Vaccine production	One country	A few countries	One or a few countries
Vaccine usage	One country or closely related countries	A few countries or closely related countries	Prioritized all over the world and first used in urgent places
Immediate benefit for disease control	Under control in one country or closely related	Under control in a few countries or closely related	Stopping international spreading
Ultimate benefit for disease control	Under control in one country or closely related	Under control in a few countries or closely related	Under control over the world
Immediate benefit for economy	Back to normal in one country or closely related	Back to normal in in a few countries or closely related	Back to normal for international business
Ultimate benefit for economy	World economy develops slowly because of pandemic in rest of the world	World economy develops slowly because of pandemic in other countries	Enhance the world economy to develop fast
Impact for future disease control	No benefit or action upon individual country	No benefit or action upon individual country or a few countries together	United international action

To promote any collaborative framework discussing prioritization in access and distribution of the vaccine, it is imperative to understand that COVID-19 is an equal threat to all human societies and cannot be contained unless globally controlled. SARS-CoV-2 has demonstrated more resilient transmission capability than other viruses associated with respiratory diseases causing a single infected individual to precipitate a COVID-19 pandemic in a very short period of time [11,12,13,14].

We as an international group of scientists call upon the world leaders to act together and develop a plan for worldwide distribution of a vaccine as opposed to multiple plans for individual countries. It is imperative that the public and government leaders understand that no single country is capable of stopping the pandemic individually and safeguard its inhabitants from repeated outbreaks, but a united front can achieve a global solution to the pandemic and, thus, prompt the recovery of the world economy.

Most importantly, the successful distribution of the vaccine in one or a few countries will not stop the pandemic and will not recover global economy (Table 1) [15,16]. It will also be a tremendous challenge to vaccinate a majority of the population in a short period of time in large countries, such as China or the US, with populations of 1.4 billion and 0.3 billion, respectively. Even if one or a few countries achieve widespread vaccination success, other factors will remain to halt the potential recovery of an economy hugely dependent on international trade [17]. Additionally, the tourism industry will not recover. Strict testing and isolation measures upon arrival would greatly reduce the number of international travelers [18,19]. This will include the number of people traveling to other countries for tourism or business, which must be greatly reduced or prohibited altogether under this vaccine distribution model. Consequently, neither tourism nor aviation industries can resume sustainable operations if the control of the epidemic remains restricted in a single or few countries [20,21].

Based on reasons mentioned above and that will be described in the subsequent paragraphs, we urge the following steps to accomplish equitable vaccine access: (1) international community to immediately start the discussion on how to form an organized approach to equitable distribution, (2) develop a vaccine distribution plan based on prioritization of needs, and (3) organize and carefully coordinate global distribution.

*However, in case the international community does not reach a consensus* on how to distribute the vaccine to achieve worldwide equitable access, at a minimum we call for a distribution plan that includes essential people for stopping disease transmission and promoting the recovery of the global economy [10]. Here we propose to vaccinate the employees in international transportation industries and international travelers. We recommend employees in the international transportation industries as the first priority for the vaccination, including international civilian aviation industry, global air freight industry, and global seaborne container trade industry [13,14,22]. These people are essential for the international business as well as for stopping international transmission of SARS-CoV-2. The total number of employees in these three groups are approximately 17 million (Figure 1).

**Figure 1:**
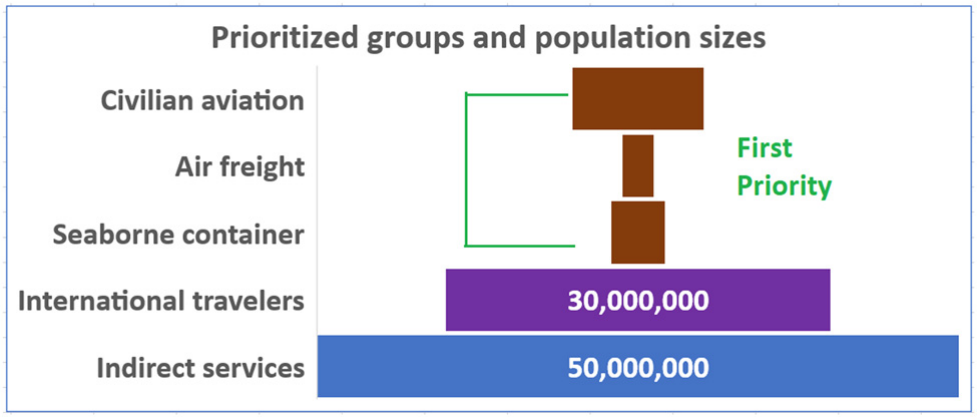
Prioritized groups and their population sizes for vaccination.

Based on the Aviation Benefits Report of International Air Transport Association [23], around 10.2 million people work in the aviation industry directly, including 2.7 million working as flight and cabin crews, executives, ground services, check-in, training, and maintenance staff; and 5.6 million working for retail, car rental, customs and immigration, freight forwarders, and catering [24].

Based on the source of Wikipedia, in 2019, the world’s three biggest international air cargo companies have a total of 1,261,000 employees (FedEx 400,000; UPS 482,100; DHL 380,000) [25]. If we assume other companies contribute about 50%, the estimated number of employees in the global air freight industry is approximately 2,500,000.

Based on the reports from the World Shipping Council, the total number of direct jobs in the shipping industry is 4.2 million [26].

Second, we recommend all international travelers receive the vaccine. Based on the world air transport statistics 2019 [27], in 2018, the total number of carried air passengers was 4,377,670,000, comprising 1,811,324,000 international passengers and 2,566,346,000 domestic passengers, increasing to over 4.5 billion passengers in 2019. However, this includes “frequent flyers,” equating to 6.5 flights per year taken by an average traveler [28]. Similarly, the Institut de Publique Sondage d'Opinion Secteur (IPSOS) report cites an average of 4.8 trips per airline traveler in 2015 [29,30]. Accordingly, the actual number of international travelers is about 400,000,000 per year. Thus, approximately 30 million people can be expected to travel internationally each month (Figure 1).

We also propose to vaccinate all people working in indirect service, such as airport hotels and restaurants for the four prioritized groups, adding a population of similar size as the total number of people in the prioritized groups [23,30].

It is important to note that the total number of people in the first three priority categories is less than 20 million. This is less than half of the people of a middle-sized country and a small portion of the total population of China or the US. Vaccination of such a count of people in China, the US or any other country will not halt the pandemic of COVID-19 and will not improve the international business and transportation. Thus, neither the stopping of COVID-19 disease nor the economy will be improved by vaccination of these individual in a country. However, if these people in international transportation are vaccinated as we proposed, the international COVID-19 transmission will be greatly halted, and the world economy will be greatly benefited. Most importantly, as long as the world is united for it, it is realistically feasible to support such an approach and produce a globally and individually much more beneficial effect than prioritizing the complete or partial vaccination of people in a single or few countries.

In conclusion, we strongly urge the international community, especially the WHO, regardless of the other considerations regarding the distribution of a potential COVID-19 vaccine, to allow the worldwide distribution of the vaccine to at least the presented priority groups and make every effort including price adjustment, shipment, resource supplement, and personal training to accomplish this target. By doing so, the world economy can begin to recover and the international spreading of the disease will be largely halted. With regard to air passengers, if vaccination becomes mandatory for travelers and the vaccine is available to them, we will be able to achieve effective infection control and regain a functional global and local economy.

This article serves as a call to the public and the leaders of the world to support such an international collaboration and put the health of all humans and world economy in front of individual short-term gain.
